# Biocompatible Inorganic PVD *Me*SiON Thin Films (*Me* = Cr or Zr) Used to Enhance the Bond Strength Between NiCr-Based Metallic Frameworks and Ceramic in Dental Restorations

**DOI:** 10.3390/dj13070318

**Published:** 2025-07-14

**Authors:** Mihaela Dinu, Cosmin Mihai Cotrut, Alina Vladescu (Dragomir), Florin Baciu, Anca Constantina Parau, Iulian Pana, Lidia Ruxandra Constantin, Catalin Vitelaru

**Affiliations:** 1 Department for Advanced Surface Processing and Analysis by Vacuum Technologies, National Institute of Research and Development for Optoelectronics—INOE 2000, 077125 Magurele, Romania; mihaela.dinu@inoe.ro (M.D.); anca.parau@inoe.ro (A.C.P.); iulian.pana@inoe.ro (I.P.); lidia.constantin@inoe.ro (L.R.C.); 2 Faculty of Materials Science and Engineering, National University of Science and Technology Politehnica Bucharest, 060042 Bucharest, Romania; cosmin.cotrut@upb.ro; 3 Department of Strength Materials, Faculty of Industrial Engineering and Robotics, National University of Science and Technology Politehnica Bucharest, 060042 Bucharest, Romania; florin.baciu@upb.ro

**Keywords:** dental restoration, NiCr, cathodic arc evaporation, oxynitrides, 3-point bending test, bond strength

## Abstract

**Background/Objectives:** The increasing demand for aesthetics in dentistry has driven significant advancements in both materials and techniques. The primary cause of ceramic detachment in dental restorations is extensive mechanical stress, which often results in detachment and clinical complications. This study aims to improve the bond strength between NiCr-based metal frameworks and ceramic coatings by introducing biocompatible inorganic *Me*SiON thin films (*Me* = Cr or Zr) as interlayers. **Methods:** *Me*SiON coatings with a thickness of ~2 μm were deposited on NiCr alloy using cathodic arc evaporation. To tailor the stoichiometry, morphology, and mechanical properties of the coatings, the substrate bias voltage was varied: −50 V, −100 V, −150 V, −200 V. Structural and surface characterization was performed using SEM/EDS, XRD, profilometry, and contact angle analysis. The coating adhesion was evaluated by using standardized scratch testing, while the bond strength was evaluated using a three-point bending test. **Results:** The NiCr alloy exhibited a dendritic microstructure, and the ceramic layer consisted mainly of quartz, feldspar, kaolin, and ZrO_2_. ZrSiON coatings showed superior roughness, elemental incorporation, and adhesion compared to Cr-based coatings, these properties being further improved by increasing the substrate bias. The highest bond strength was achieved with a ZrSiON coating deposited at −200 V, a result we attributed to increased surface roughness and mechanical interlocking at the ceramic-metal interface. **Conclusions:** CrSiON and ZrSiON interlayers enhanced ceramic-to-metal adhesion in NiCr-based dental restorations. The enhancement in bond strength is primarily ascribed to substrate bias-induced modifications in the coating’s stoichiometry, roughness, and adhesion.

## 1. Introduction

**Figure 1:**
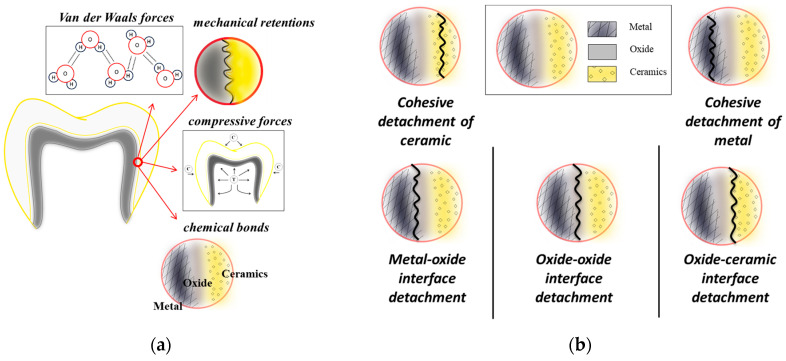
Physical and chemical factors that influence the metallic-ceramic bond strength in dental restorations (**a**) and the classification of ceramic detachment types (**b**).

In recent years, there has been an increasing global demand for dental treatments. As a result, new solutions and materials have been developed to enable the reconstruction of natural teeth in terms of both function and aesthetics. In dental restorations, the most important requirement is the adhesion between the metal and ceramic parts, their interaction being dependent on several physical and chemical factors (Figure 1a) [1]. Bonds of a physical nature are formed when the metal surface is covered by the ceramic mass, a mechanism supported by the molten state of the ceramic, vacuum heating, and optimal processing. Additionally, *Van der Waals* forces and the proportional ratio between the contact surface and the size of the layer formed at the interface have a direct influence [2]. Other contributing factors involve the compatibility between the thermal expansion of the two materials and the resulting stresses. Ideally, the contraction of the alloy needs to be slightly greater than that of the ceramic. On the other hand, chemical bonds form as a result of the interlocking molecules of the two structures that come into contact. Moreover, an oxide layer is created by adding special oxide-generating agents that diffuse to the metal surface during oxidation heat treatment. This process leads to the migration of ions from the alloy surface toward the ceramic layer, resulting in the formation of ionic bonds [3].

It is well known that in the oral environment, crowns and dental bridges can be subjected to different mechanical loads, like traction, compression, or shear stresses. There are several types of failure depending on the nature of the formed interfaces (Figure 1b). The debonding location can provide useful information about the adherence mechanism [4]. Usually, the fractures within the ceramic component can indicate high bond strength, while those at the oxide-oxide interface or metal-oxide can be evidence of poor bond strength between metal and ceramic. However, the predominant type of failure depends on the metal surface treatment, the compatibility of the used materials, or the thermal treatment procedures [1,5,6].

As observed in previous studies, less attention has been paid to the surface modification of NiCr-based dental restorations. While the use of CoCr alloy is well documented in the literature and has advantages, such as corrosion resistance and biocompatibility [7,8,9,10], NiCr alloy remains widely used in removable dentures, especially for patients without nickel allergies. Its advantages are mainly related to cost effectiveness and favorable mechanical properties, since many technicians often prefer a material that is easier to handle during complex laboratory procedures and offers an issue-free casting process [11]. Even if the NiCr-ceramic degree of fit is considered, there is a degree of fragility of the bond strength, since the two components have completely different structures. To date, continuous efforts have been made to optimize the interface and increase the efficiency of this bond. For this purpose, different surface treatments have been considered, including oxidation, sandblasting, chemical/electrochemical etching, and even laser treatments [6,12,13,14]. In a previous report, when TiSiN coatings obtained by the cathodic arc method were used as interlayers, the metal-ceramic bond strength reached ~35 MPa, compared with only ~32 Mpa obtained for bare NiCr-based systems [15]. The hypothesis tested was that TiSiN coatings exhibit suitable properties for dental applications and the bias voltage used in this case can have a beneficial influence.

In the present study, we further explore the addition of transition thin films to NiCr-based frameworks to improve the overall metal-ceramic bond strength. Apart from selecting different transition elements, in order to contribute to a higher compatibility degree, a high oxygen content was added to achieve an oxynitride structure with a ceramic character. Therefore, in the present experiment, *Me*SiON monolayers (*Me* = Cr or Zr) were obtained via reactive cathodic arc deposition. The influence of each coating type was assessed by varying the substrate bias voltage during deposition. The present study is based on the hypothesis that the application of biocompatible inorganic *Me*SiON (*Me* = Cr, Zr) thin films, combined with an increased substrate bias voltage, will enhance the bond strength between NiCr-based metal frameworks and dental ceramic coatings.

The main properties which influence the bond strength were investigated for each part of the overall system: the metallic framework, the proposed coatings, and the dental ceramic. Elemental and structural analyses were performed, as well as surface roughness, wettability, and adherence to the substrate. Additionally, a mechanical 3-point bending test was applied for the metal-thin film-ceramic characterization, and the resulting surfaces were analyzed according to interfacial detachment types.

## 2. Materials and Methods

**Figure 2:**
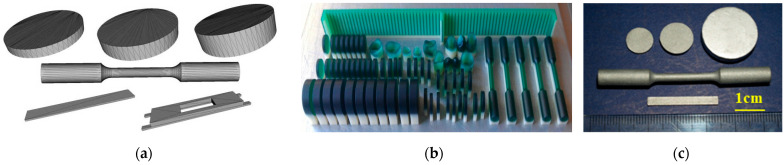
3D models designed in SolidWorks (**a**), printed polymeric patterns (**b**) and casted experimental samples (**c**).

The NiCr alloy specimens were obtained using the lost wax technique, following conventional laboratory techniques and based on the manufacturer’s specifications. First, the polymeric patterns were designed in SolidWorks (version 2010) with the specific dimensions needed for each investigation, then manufactured using a 3D printer (ProJet DP3000, 3D Systems, Rock Hill, SC, USA). Further, Argeloy NP alloys (Argen, San Diego, CA, USA) were melted and cast using an induction furnace with centrifugal force (Ducatron Serie 3, Ugin Dentaire, Seyssinet-Pariset, France) (Figure 2). The type, dimensions, and investigations of the designed experimental samples are presented in Table 1. The NiCr specimens were sandblasted with 150 μm alumina particles to remove debris and enhance roughness. The purpose of surface conditioning was to increase surface energy and wettability.

The *Me*SiON coatings (*Me* = Cr or Zr) were deposited using the cathodic arc evaporation technique by using a *Me*Si cathode (84 at.% *Me*, 16 at.% Si) in a (N_2_ + O_2_) mixed atmosphere. The samples were mounted on a rotating substrate holder and were sputter-etched with 1000 eV Ar^+^ for 300 s. The main process parameters were: 6 × 10^−3^ Pa total gas pressure, 120 sccm N_2_ flow rate, 80 sccm O_2_ flow rate, and 90 A arc current. The substrate bias voltage (V_b_) was adjusted to −50 V, −100 V, −150 V, and −200 V, thus obtaining four coatings for each type. The previously mentioned parameter was varied to evaluate the correlation between the coating properties and the deposition conditions and to identify the optimal solution needed for improving the metal-ceramic adhesion.

The ceramic was sintered on NiCr plates with the dimensions specified in the ISO 9693:2000 standard [18]. In the current study, Vision Classic type ceramic (Wohlwend AG, Liechtenstein) was selected, conventionally used for NiCr alloys, with an expansion coefficient of about 13.8–15.2 μm/mK (25–500 °C). For bonder, opaque, dentin, and glaze firing, a computer-controlled dental ceramic furnace was used (JELRUS VIP Universal, Air Techniques, Inc., New York, NY, USA).

The elemental composition of both the NiCr alloy and the sintered ceramic was investigated using an energy dispersive X-ray spectrometer (Bruker, Billerica, MA, USA). To study the surface morphology, a scanning electron microscope was used (Hitachi TM3030 Plus, Tokyo, Japan). The elemental composition of the coatings was investigated by glow discharge optical emission spectroscopy using a SPECTRUMA GDA-750HP instrument (Spectruma Analytik GmbH, Hof, Germany).

The phase composition was investigated using an X-ray diffractometer (Miniflex II, Rigaku, Tokyo, Japan) with CuK_α_ radiation (λ = 0.15405 nm). The surface roughness was evaluated using a Dektak 150 profilometer (Bruker, Billerica, MA, USA). For this purpose, the measurements were performed over a distance of 4 mm for 200 s using a 2.5 µm radius stylus.

The wettability was analyzed by the sessile drop method, using an optical tensiometer (Attension Theta Lite 101, Biolin Scientific AB, Göteborg, Sweden) in atmospheric conditions (25 °C, 40% humidity). The contact angles between the test liquid (a solution based on propylene glycol used for mixing the ceramic powder) and the surface of the specimens were measured.

The adhesion of the coatings to the NiCr alloy substrate was investigated by scratch test according to the BS EN 1071-3:2005 standard [19] and using a Bruker’s Universal test system UMT TriboLab™ (Bruker, Billerica, MA, USA). SEM images were recorded in order to identify the load at which the delamination of the coating occurred as a function of distance, and thus to determine the critical load parameter (L_c_).

Metal-thin film-ceramic characterizations were made according to the ISO 9693:2000 standard [18]. Prior to bond strength evaluation, six metal specimens were tested for Young’s modulus estimation (ISO 6892-1:2019 [16]). The debonding/crack-initiation strength (*τ_b_*) was calculated using Equation (1):

*τ_b_* = F_fail_ × k (1)

where F_fail_ = the fracture force and k = constant based on the metal thickness and Young׳s modulus. Additionally, micrographs of the metal—coating—ceramic interface were acquired to gain insight into different aspects involved in the debonding process.

All of the materials and equipment used in the current study are described in Table 2.

## 3. Results

### 3.1. NiCr Dental Alloy Characterization

#### 3.1.1. Surface Morphology and Elemental Composition

**Figure 3:**
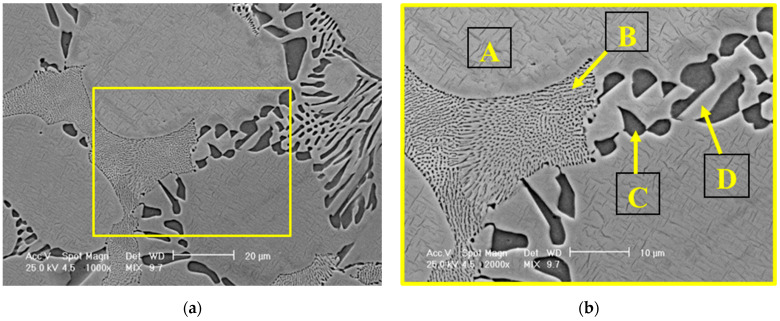
(**a**) SEM micrographs of NiCr alloy microstructure at 1000× magnification and (**b**) at 2000× magnification. The marked areas A, B, C, and D were selected for EDS analysis.

The micrographs of the NiCr alloy (Figure 3) indicated a dendritic microstructure typical of cast alloys [20,21,22], consisting of a primary phase of solid solution (A) and two types of eutectic phase with a lamellar appearance: a fine (B) and a coarse eutectic (C, D). Whereas the fine lamellar eutectic phase is formed as a result of rapid local solidification, the coarse eutectic phases (with larger lamellar spacing) usually form in the regions where cooling rates are slower. Mathieu et al. [23] discovered that the formation of fine precipitates, such as carbides and intermetallic phases, has multiple benefits such as strengthening the alloy, improving ductility, and improving mechanical performance. Their size, distribution, and morphology can be slightly changed after the heat treatment in accordance with their chemical composition [24]. However, the optimization of precipitate distribution further enhances mechanical properties by reducing elemental segregation. The mentioned refinement mechanism can be considered a greatly important benefit in dental restorations [25]. Apart from high strength and hardness, corrosion resistance is critical to preventing the degradation of dental materials in the oral environment [26,27].

Based on the EDS analysis presented in Table 3, the solid solution phase was primarily composed of nickel (Ni) and chromium (Cr), with minor additions of molybdenum (Mo) and aluminum (Al). In contrast, the fine eutectic structure exhibited a higher Mo content, which appears to be at the expense of other constituent elements. Regarding the coarse eutectic structure, the identified compounds were predominantly formed from Ni, while the lamellar structures within this region displayed a composition comparable to that of the solid solution.

#### 3.1.2. Phase Composition

**Figure 4:**
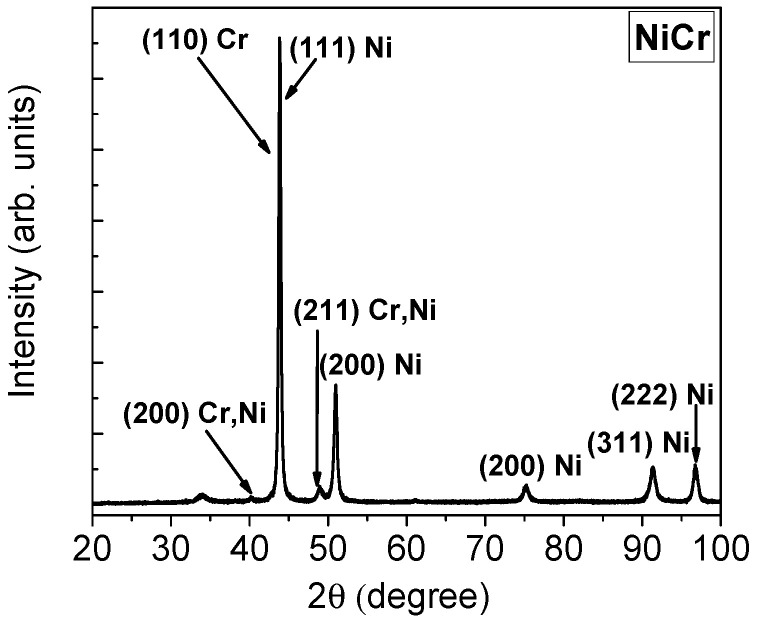
XRD pattern of NiCr alloy.

The results of the X-ray diffraction analysis for the NiCr alloy are shown in Figure 4. The diffraction peaks corresponding to the Ni element (with a face-centered cubic structure) and the crystallographic planes (111), (200), (311), (222), and (JCPDS 1-1258) were identified. Notably, the diffraction peaks observed at 2θ = 44.4° arose from the superposition of the reflections attributed to the (111) plane of Ni and the (110) plane of Cr (body-centered cubic structure, JCPDS 6-0694). Additional reflections at 2θ = 39.3° ((200) plane) and at 2θ = 48.9° ((211) plane) provided clear evidence for the formation of a Cr-Ni solid solution (JCPDS 26-0429).

### 3.2. Thin Films Characterization

#### 3.2.1. Surface Morphology and Elemental Composition

**Figure 5:**
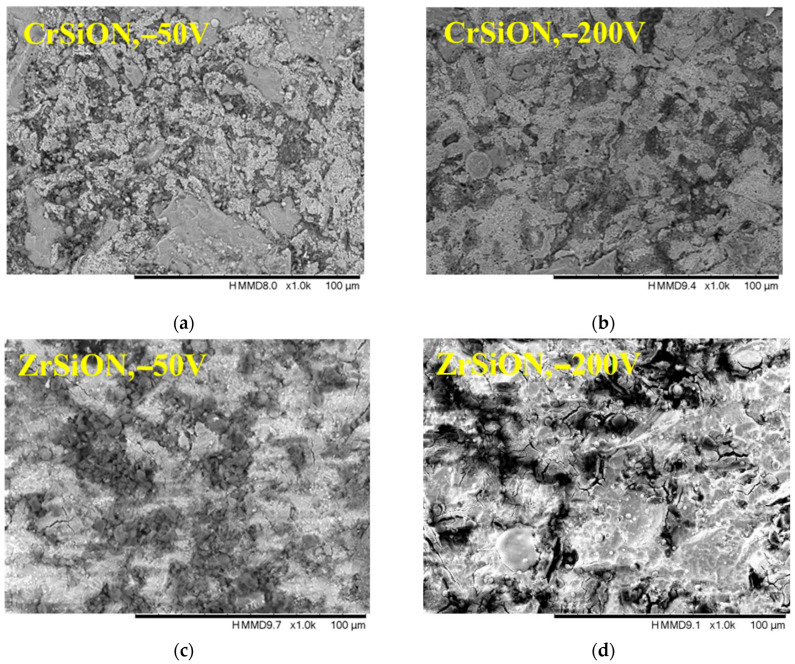
SEM micrographs of CrSiON (**a**,**b**) and ZrSiON coatings (**c**,**d**) as a function of substrate bias voltage.

Selected mixed backscattering and secondary electron micrographs of *Me*SiON coatings (at ×1000 magnification) are presented in Figure 5. As observed, all the coated surfaces appeared rough and granular, indicating that the initial conditioning treatment applied before deposition (i.e., 150 μm alumina particle sandblasting) had a significant influence on the final appearance. As stated before, there are multiple studies that report surface conditioning treatments to promote surface roughness [6,13]. In the current study, a synergistic physical-chemical effect was expected. Therefore, the bond strength was improved by providing both mechanical interlocking and proper conditions for a uniform coating growth, which minimized the metal-porcelain mismatch. Their performance was further enhanced by tailoring the deposition conditions—in this case, the substrate bias. Even though there was no clearly visible compactness as a function of substrate bias, no significant difference was observed among the presented SEM micrographs, and it is well known that higher ion energy leads to an increased density and surface diffusion [28].

**Figure 6:**
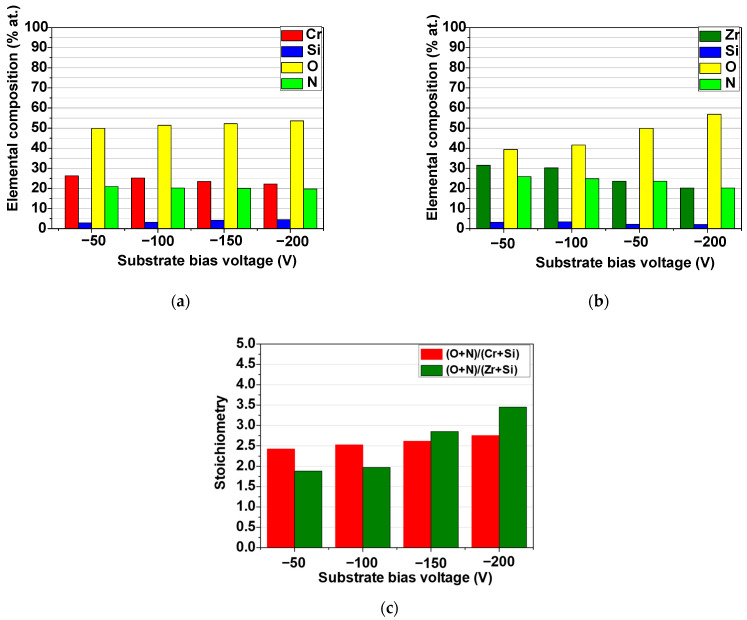
Elemental composition for CrSiON (**a**) and ZrSiON coatings (**b**); (**c**) stoichiometry of the investigated coatings as a function of substrate bias voltage.

The elemental composition of the *Me*SiON coatings was given in Figure 6a,b, confirming the ceramic nature of oxynitride structures. Indeed, one may notice that for all layers, oxygen was found to be ~40 at.% at −50 V and increased with negative substrate bias, reaching ~57 at.% at −200 V, which was detrimental to the other elements. Since this tendency was more evident for Zr-based coatings, it is reasonable to consider a higher degree of fit and porcelain compatibility in this case. The calculated (O + N)/(*Me* + Si) ratio (given in Figure 6c) confirmed the intended suprastoichiometry, which varied between ~2 and even ~3 for ZrSiON obtained at −200 V, further supporting the previous idea. The obtained results demonstrated that nonmetallic elements made a major contribution compared to the metallic ones. It is well known that oxygen is more reactive than nitrogen, and its fraction increases due to the so-called poisoning of the target phenomenon, an effect which can be beneficial in the current study. Although the oxide formation on the surface has a low deposition rate and the material ejection becomes difficult, it can tailor the coating composition toward improved functional properties (ex: thermal stability and enhanced corrosion resistance) [29].

#### 3.2.2. Roughness

**Figure 7:**
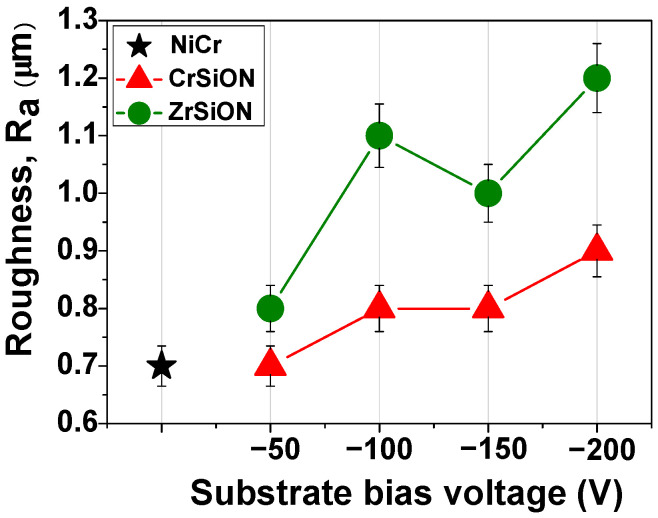
The evolution of the surface roughness of the investigated *Me*SiON coatings as a function of substrate bias voltage.

Surface roughness results are presented in Figure 7. The measurements were performed for CrSiON and ZrSiON coatings, as well as for the uncoated NiCr alloy, which served as the control. For each substrate bias voltage applied (−50 V, −100 V, −150 V, −200 V), a total of five specimens per group (n = 5) were analyzed. The results are presented as mean values accompanied by their corresponding standard deviations. One may notice relatively similar values of CrSiON coatings deposited at V_b_= −50 V compared to the bare substrate (~0.7 µm), whereas a slightly higher initial value was exhibited by Zr-based oxynitride (~0.8 µm). Overall, there was a general increasing trend in the average roughness parameter R_a_ as a function of negative substrate bias voltage. Even though CrSiON coatings proved to be less sensitive to bias-induced surface roughening, reaching a slight increase (~0.9 µm) at −200 V, there was still an improvement compared to the uncoated NiCr substrate. Adding a functional layer imparts benefits apart from roughness, since biasing does not compromise coating quality. Moreover, it promotes adatom mobility that leads to a denser surface, as indicated by previous AFM studies [30]. On the other hand, the ZrSiON coating was more susceptible to variations in bias voltage during plasma deposition, reaching ~1.2 µm at −200 V. Similar results were reported by Niu et al., who attributed the mentioned effect to the direct correlation of ion energy and adatom mobility [31]. As a consequence, both the quantity of the formed clusters and the packing density increased [32].

#### 3.2.3. Wettability

**Figure 8:**
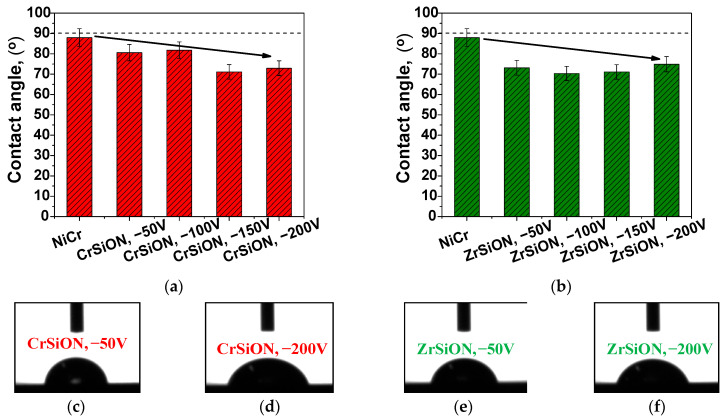
Contact angle values of investigated specimens (**a**,**b**) and images of the test liquid droplet on the surface of selected *Me*SiON coatings as a function of substrate bias voltage (**c**–**f**).

The contact angle values of a propylene glycol-based solution on the investigated surfaces are presented in Figure 8. Wettability measurements were conducted for CrSiON and ZrSiON coatings as well as for the uncoated NiCr alloy, which served as the control. For each applied substrate bias voltage (−50 V, −100 V, −150 V, −200 V), three specimens per group (n = 3) were analyzed. The results are expressed as mean values ± standard deviation. Representative images of the test liquid droplets on the sample surfaces are also included. As observed, both sets of coated specimens exhibited more hydrophilic surfaces compared to the substrate. As previously suggested, these surfaces are highly recommended for biomedical applications [33,34,35,36]. Moreover, a hydrophilic surface facilitates better interface contact and promotes a more efficient ceramic coverage and a better ceramic-metal adhesion. The values obtained can be linked with the topography of the proposed surfaces as well as the high oxygen content, which was found in the current study to be in ~40–57 at.% range.

#### 3.2.4. Adhesion to the NiCr Substrate

**Figure 9:**
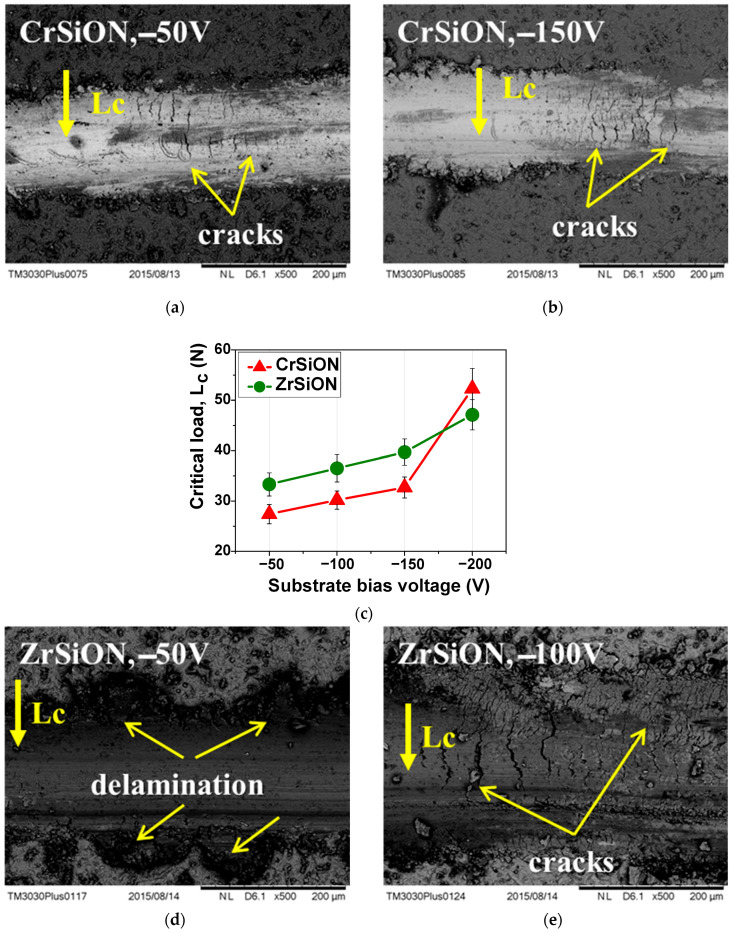
SEM micrographs showing features of failure mechanisms of selected *Me*SiON coatings (**a**,**b**,**d**,**e**) and the evolution of critical load as a function of substrate bias voltage (**c**).

The mechanism of coating failure can be cohesive (failure occurs within the same phase) or adhesive (failure occurs within different phases). Since cracks due to cohesive failure usually appear before complete delamination at the coating–substrate interface, this phenomenon was considered as an early stage of failure. At this point, the critical load (Lc) was used as an adhesion indicator for visible cohesive cracks/partial spallation. Although researchers distinguished among multiple critical loads (i.e., Lc1, Lc2, or Lc3), the first cohesive cracking was commonly defined as the adhesion strength of the coating [37,38]. Additionally, features associated with failure mechanisms were highlighted by SEM micrographs of selected coatings (Figure 9a,b,d,e).

Adhesion was measured for the CrSiON and ZrSiON coatings and NiCr alloy (control), with n = 3 specimens per group for each bias voltage applied (−50 V, −100 V, −150 V, −200 V). Results were reported as mean ± standard deviation (Figure 9c). As expected, the evolution of the Lc parameter characteristic of the investigated specimens indicated an increase in the coatings’ critical load as a function of negative substrate bias voltage. SEM micrographs revealed both delamination and the presence of cracks, typically caused by internal stresses within the coating. Despite a partial failure appearance, most of the coatings maintained their integrity up to ~30 N. This behavior can be essential to the overall bond strength, as higher adhesion to the substrate also influences the system’s overall mechanical performance.

### 3.3. Dental Ceramic Characterization

#### 3.3.1. Surface Morphology and Elemental Composition

**Figure 10:**
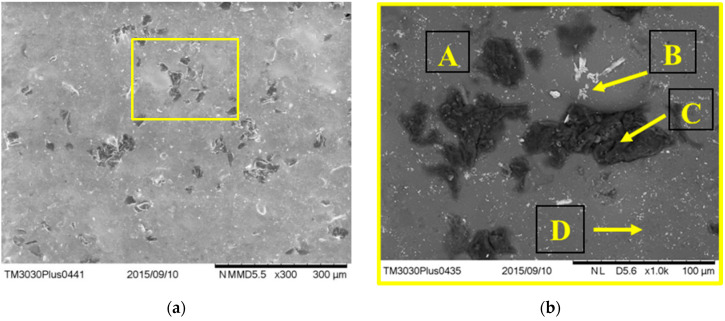
Micrographs of the ceramic component after sintering at 300× (**a**) and 1000× (**b**) magnification. The marked areas of representative A, B, C, D correspond to the selected EDS investigated by areas.

Mixed backscattering and secondary electron micrographs of the ceramic component after sintering are presented in Figure 10, both at 300× and 1000× magnifications. An overall surface investigation showed heterogeneous regions with microstructural differences. A more detailed observation enabled the examination of different components, marked as A, B, C, and D. In order to have an indication of the elemental composition of the mentioned areas, an additional EDS investigation was performed.

The EDS results (Table 4) showed that the constitutive elements of the investigated ceramic were identified as O, Si, K, and Al, which correspond to the main components of dental porcelain: quartz (SiO_2_), feldspar (KAlSi_3_O_8_), and kaolin (Al_2_O_3_*2SiO_2_*2H_2_O). The high oxygen content (~48 at.%), silicon (~21 at.%), and carbon (~15 at.%) found in area A suggested a silica-rich glassy phase, commonly used for aesthetic appearance [39]. Also, the presence of the Ba element in the B area proved the use of BaO flux in the composition of the investigated ceramic. Note the high carbon content (~62 at.%) of the dark-colored compounds (C), probably due to SiC added as reinforcement particles for enhanced mechanical properties. Additionally, the presence of Zr was also identified in area (D), usually added in dental ceramics as ZrO_2_ for an opaque appearance [40]. Aside from the main compounds, several fluxes were added to lower the sintering temperature (i.e., Na_2_O and K_2_O).

#### 3.3.2. Phase Composition

**Figure 11:**
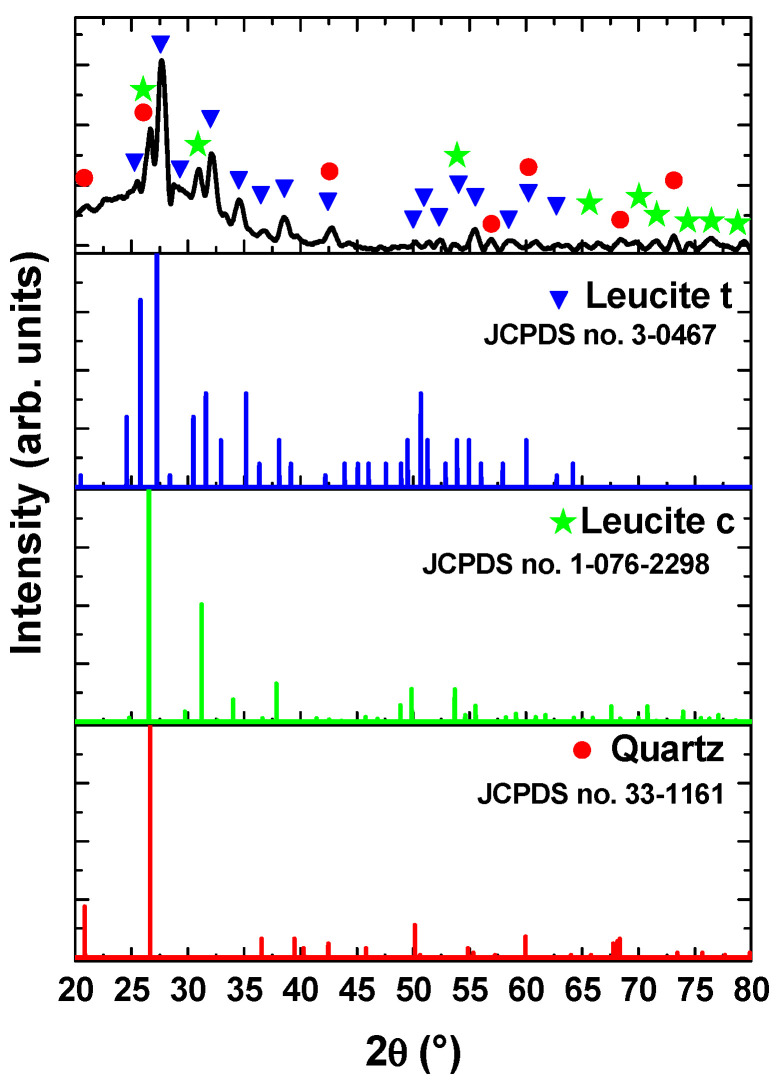
XRD pattern of the ceramic component.

The XRD pattern of the investigated ceramic is presented in Figure 11. The results indicated the presence of the crystalline leucite phase (KAlSi_2_O_6_), which has an important role in controlling the coefficient of thermal expansion of ceramic in order to match with the metal framework, thus minimizing crack formation during the sintering process [41,42]. As indicated in the literature, the crystalline structure of the leucite phase varied as a function of sintering temperature, and its amount depended on the cooling rate [43]. In the present study, the XRD analysis indicated the predominant crystallization of the tetragonal leucite phase (JCPDS no. 3-0467), but some peaks (with relatively lower intensity) were also assigned to the leucite phase with a cubic structure (JCPDS no. 1-076-2298). This result was attributed to the transformation of the cubic structure of the leucite into a tetragonal phase at temperatures of about 620–625 °C [44]. Moreover, quartz phases with hexagonal structures were also identified according to JCPDS no. 33-1161.

### 3.4. Metal-MeSiON-Ceramic Characterization

#### 3.4.1. 3-Point Bending Test

**Figure 12:**
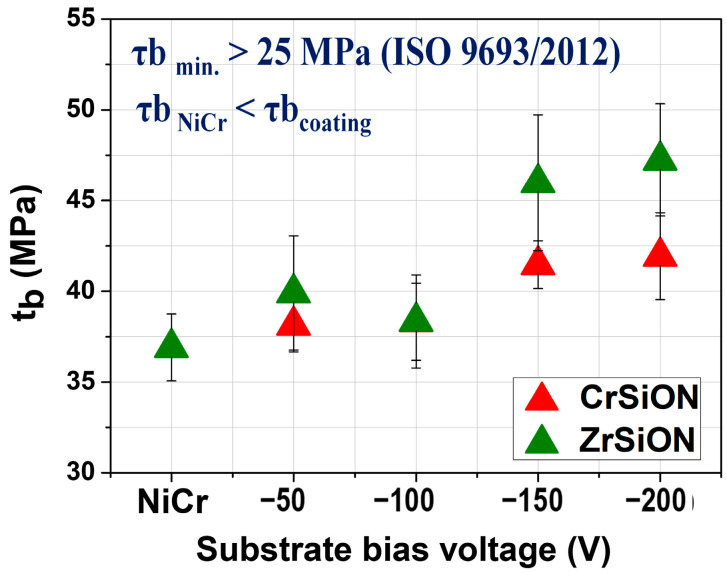
The bond strength evolution of metal-*Me*SiON-ceramic systems as a function of substrate bias voltage.

Following ceramic deposition, bond strength measurements were carried out for CrSiON and ZrSiON coatings, as well as for the uncoated NiCr alloy that served as the control. For each substrate bias voltage applied (−50 V, −100 V, −150 V, and −200 V), five specimens per group (n = 5) were tested. The results are presented as mean values ± standard deviation, as illustrated in Figure 12. The results of the three-point-bending test for the systems where *Me*SiON coatings were used indicated an increasing tendency of the debonding/crack-initiation strength parameter (t_b_) according to the negative substrate bias. Note that all of the values exceeded 25 MPa, which is the minimum requirement for bond strength specified by the ISO 9693:2000 standard [18]. Additionally, the systems where the coatings were used showed higher values compared to the control ones, mainly due to a more hydrophilic character exhibited by the coated surfaces. In this case, the obtained high values can be explained by a synergetic effect of chemical and physical factors, as follows. Based on previous observations, the elemental composition had an important contribution, since the oxygen content was directly related to deposition conditions, leading to over-stoichiometry of the obtained coatings (especially higher (O + N)/(Me + Si) ratio for ZrSiON).

On the other hand, the surface roughness and therefore the coatings-substrate adhesion can also contribute to the bond strength of the investigated systems, with results influenced by a mechanical interlocking process. Although the *Me*SiON coatings exhibited an amorphous structure when deposited at a lower substrate bias (i.e., −50 V, detailed microstructural study published in Ref. [28]), structural defects (microdroplets, cracks, and pores) were observed. In contrast, the coatings obtained at −200 V were more densely packed and had a defect-free structure, leading to a better adherence to the substrate and consequently a higher bond strength [28]. As stated earlier, the mentioned morphology differences of the obtained coatings may be due to adatom mobility of the condensed species during deposition [31].

#### 3.4.2. Surface Morphology of Debonded Plates

**Figure 13:**
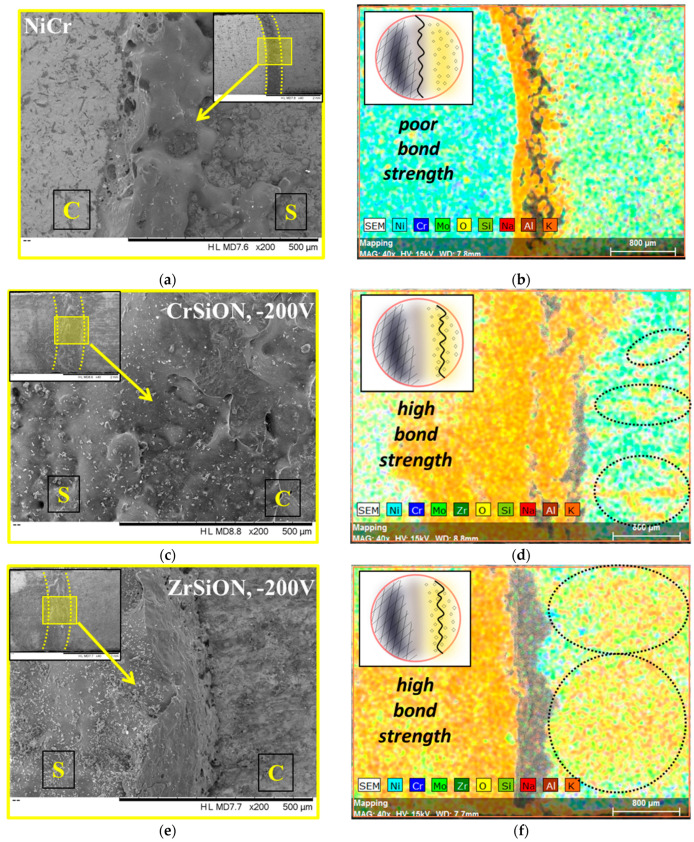
SEM images (**a**,**c**,**e**) and EDS mapping analysis (**b**,**d**,**f**) of the metal-thin film-ceramic systems after 3-point bending test (S = *Me*SiON-coated or uncoated NiCr plate, C = area where ceramic layer was bonded) (the inset presented in each SEM image was recorded at ×40 magnification).

Following the mechanical loading required by the 3-point bending test, SEM micrographs and corresponding EDS mapping images of the fracture surfaces after debonding were recorded in order to assess interfacial failure modes (Figure 13). For comparison purposes, only the best-performing coatings were selected (i.e., CrSiON and ZrSiON obtained at −200 V) with uncoated NiCr-based plates used as controls. The dashed line presented in the inset (×40 magnification) separates the *Me*SiON-coated or uncoated NiCr area (marked with S) from the area where the ceramic layer was bonded (marked with C). Additionally, a higher magnification image (×200 magnification) highlighted the interface region where the debonding occurred (Figure 13a,c,e). To properly identify the interfacial failure modes, EDS mapping analysis was also performed (Figure 13b,d,f).

Note that for the control sample, the fracture area appeared smoother, indicating an oxide-oxide interface detachment failure mode, since only elements specific to the oxidized substrate were found in this area where the ceramic component was sintered (C) (Figure 13b). On the other hand, the morphology of the *Me*SiON-coated plate after ceramic detachment indicated a similar fracture pattern in both cases, with the ceramic associated features evenly distributed (Figure 13c,e). Residues of ceramic were found via EDS in the area where the ceramic layer was sintered, being an indication of a cohesive ceramic detachment (Figure 13d,f). The elemental distribution pattern indicated that fracture occurred within the ceramic component, the specific elements being more pronounced when a ZrSiON thin film was used (larger marked area). The more uniform bonded ceramic appearance in this case represented a validation of the previous results of the mechanical 3-point bending test, in which a ~28% improvement was found for ZrSiON obtained at −200 V, over only ~14% improvement for CrSiON obtained under the same conditions.

## 4. Discussion

This study presents a mechanical evaluation of the bond strength of NiCr-based metallic frameworks coated with PVD (Cr,Zr)SiON interlayers at the interface with sintered ceramic. The investigation encompassed a comprehensive analysis of each component, as well as three-point bending tests of the assembled system conducted in accordance with the ISO 9693:2000 standard [18]. Particular emphasis was placed on the deposition of approximately 2 µm thick Cr- and Zr-based oxynitride coatings via cathodic arc evaporation. The deposition parameters, specifically the substrate bias voltage (set at −50 V, −100 V, −150 V, and −200 V), were systematically varied to obtain ceramic-like structures with enhanced mechanical and chemical properties. The results demonstrated a bias-induced surface roughening effect, which facilitated improved adhesion and reduced contact angles, thereby promoting more effective ceramic coverage. The higher increment of Ra observed in the case of ZrSiON compared to CrSiON under the same conditions was attributed to material-specific factors such as atomic mass, since heavier atoms (Zr > Cr atomic mass) further contribute to surface modifications during the re-sputtering process [45,46]. Additionally, the direct correlation of the increasing trend of Lc and deposition conditions applied was linked with the more defect-free structure (i.e., lack of cracks, voids, and microdroplets) of the coatings obtained at higher V_b_ [28]. Moreover, the differences in the adhesion behavior of the investigated systems were due to the heat of mixing (ΔH_mix_) of the main metallic component elements of both the oxynitride coatings and the uncoated NiCr specimen, since ΔHmix Zr-Ni= −63 kJ/mol > ΔHmix Cr-Ni= −7 kJ/mol [47]. Regarding the values obtained at −200 V, there is no clear consensus supporting the latter assumption. However, considering that the error bars are overlapping, it is reasonable to consider similar adhesion behavior of *Me*SiON systems and the involvement of other physical factors, such as local roughness fluctuation.

Regarding wettability, as data from the literature have demonstrated, the presence of a high oxygen content promoted the formation of polar functional groups, which led to lower contact angle values [48]. Even though a rougher surface typically enhances surface wettability, the applied operating conditions had a minimum effect in this case, as increasing the bias voltage led only to minor improvements. In contrast to the general trend observed between wettability and bias voltage variation, a slightly higher average contact angle value was recorded for the ZrSiON coating deposited at −200 V. This deviation may be attributed to a combination of material-specific properties and the surface chemistry/morphology that occur during the deposition process. While coating densification typically enhances surface performance, the resulting reduction in porosity may limit the penetration of the test liquid droplet [49]. Furthermore, when the bias voltage exceeds an optimal threshold, the excessive ion energy can induce a nano-scale texture or specific patterning, potentially promoting partial Cassie–Baxter wetting behavior [50,51]. Therefore, achieving an optimal balance between ion energy and film growth dynamics is essential. However, by taking into account the error bar ranges across all Zr-based coated surfaces, the apparent increase at −200 V may not be statistically significant. An overall trend of increasing desirable properties with higher substrate bias voltage was observed and attributed to a denser, defect-free coating microstructure at elevated bias (notably −200 V), confirming previous results [28].

The debonding/crack-initiation strength values obtained for systems incorporating *Me*SiON coatings were higher than those of uncoated NiCr-based plates. This enhancement was attributed to the coatings’ more hydrophilic surfaces with favorable stoichiometry ((O + N)/(Me + Si) ≈ 2–3), as well as improved coating–substrate adhesion that supported mechanical interlocking at the interface. Scanning electron microscopy (SEM) and elemental mapping analyses conducted to assess failure modes revealed that fractures predominantly occurred within the ceramic component when *Me*SiON coatings were applied, indicating superior bond strength. Moreover, a greater ceramic coverage area was observed following debonding for the Zr-based monolayers, corroborating the bond strength improvements measured (~28% for ZrSiON at −200 V compared to ~14% for CrSiON at the same bias).

### Limitations

This study demonstrated the potential of CrSiON and ZrSiON coatings to enhance the bond strength between NiCr-based metal frameworks and sintered ceramics, as evidenced by detailed mechanical and structural characterization. However, several limitations must be acknowledged:

- In vitro conditions: All experiments were conducted under controlled in vitro laboratory conditions, which do not fully replicate the complex physiological environment of the oral cavity. Critical factors such as the presence of saliva, fluctuating pH levels, thermal cycling, and mechanical fatigue were not simulated, although they significantly influence the long-term clinical performance of the proposed coatings.
- Absence of long-term performance evaluation: While initial bond strength was assessed using standardized mechanical tests according to ISO 9693:2000 [18], the study did not include evaluations of long-term durability, fatigue resistance, or aging behavior. Parameters such as thermocycling, corrosive degradation, and repetitive loading were not addressed, thus limiting the predictive capacity regarding the coatings’ performance over time in the clinical environment.
- Lack of biological testing: Although CrSiON and ZrSiON coatings are considered biocompatible based on their composition and supporting literature, no experimental assessments of cytotoxicity, cell adhesion, or other biological tests were performed in the present study. Therefore, the biological safety and compatibility of these coatings with oral tissue remain to be experimentally assessed. Future studies should include comprehensive biological evaluations to validate the clinical applicability of these coatings.

## 5. Conclusions

Based on the experimental findings, the main conclusions of this study are summarized as follows:

- Thin films of (Cr,Zr)SiON deposited by cathodic arc evaporation significantly improved the bond strength at the metal–ceramic interface in dental restorations.
- Increasing the substrate bias voltage, particularly to −200 V, resulted in denser and more uniform coatings, which exhibited enhanced surface roughness, adhesion, and hydrophilicity.
- ZrSiON coatings demonstrated a superior performance, as evidenced by a higher ceramic coverage area after debonding and an approximate 28% increase in bond strength at −200 V, compared to a ~14% improvement for CrSiON under the same conditions.

In conclusion, the incorporation of *Me*SiON (*Me* = Cr or Zr) interlayers between ceramic coatings and NiCr-based metal frameworks represents a promising strategy for improving interfacial bonding. This approach may help reduce the mismatch between metal and porcelain, thereby enhancing the durability and clinical longevity of dental restorations.

## Figures and Tables

**Table 1:** The type, dimensions, and investigations of the designed experimental samples.

Sample	Dimensions	Type of Investigations
Specimens 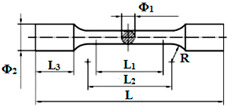	Φ_1_ = 3 ± 0.1 mm; Φ_2_ = 6 mm L = 42 mm; L_1_ = 15 ± 0.1 mm; L_2_ = 18 ± 0.1 mm; L_3_ = 8.5 mm	Mechanical tensile tests for determining Young’s modulus according to ISO 6892 [16]
Plates 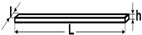	L = 25 ± 1 mm l = 3 ± 0.1 mm h = 0.5 ± 0.05 mm	3-point bending tests for determining ceramic-to-metal adhesion according to ISO 9693/2012 [17]
**Disc_1_** 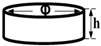	Φ = 20 mm h = 5 mm	Surface morphology and elemental composition
**Disc_2_** 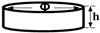	Φ = 25 mm h = 2 mm	Phase composition, Adhesion
**Disc_3_** 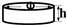	Φ = 12 mm h = 2 mm	Roughness, Wettability

**Table 2:** Materials and equipments.

Materials/Machines	Brand Name	Manufacturer Details	Composition of Materials
NiCr alloy	Argeloy NP	Argent, San Diego, CA USA	76 wt. % Ni, 14 wt. % Cr, 6 wt. % Mo, 2 wt. % Al, 1.8 wt. % Be
CrSi cathode	-	Cathay Advanced Materials Ltd., Huizhou, Guangdong Province, China	84 at.% Cr, 16 at.% Si
ZrSi cathode	-	Cathay Advanced Materials Ltd., Huizhou, Guangdong Province, China	84 at.% Cr, 16 at.%
Ceramic	Vision Classic	Wohlwend AG, Fürstentum, Liechtenstein	-
3D printer	ProJet DP3000	3D Systems, Rock Hill, SC, USA	-
Induction furnace with centrifugal force	Ducatron Serie 3	Ugin Dentaire, Seyssinet-Pariset, France	-
Computer-controlled dental ceramic furnace	JELRUS VIP Universal,	Air Techniques, Inc., New York, NY, USA	-
Energy dispersive X-ray spectrometer	-	Bruker, Billerica, MA, USA	-
Scanning electron microscope	TM3030 Plus	Hitachi, Tokyo, Japan	-
Glow discharge optical emission spectroscopy	SPECTRUMA GDA-750HP	Spectruma Analytik GmbH, Hof, Germany	-
X-ray diffractometer	Miniflex II	Rigaku, Tokyo, Japan	-
Profilometer	Dektak 150	Bruker, Billerica, MA, USA	
Optical tensiometer	Attension Theta Lite 101	Biolin Scientific AB, Göteborg, Sweden	
Scratch platform	UMT TriboLab™	Bruker, Billerica, MA, USA	

**Table 3:** Elemental composition (at.%) of NiCr alloy microstructure.

Element	Solid Solution (A)	Fine Eutectic (B)	Compounds Within the Coarse Eutectic Structure (C)	Lamellas Within the Coarse Eutectic Structure (D)
Al	6.43	4.30	-	5.19
Mo	3.31	7.94	-	4.09
Cr	16.78	15.85	2.46	16.33
Ni	73.48	71.92	97.54	74.39

**Table 4:** Elemental composition (at.%) of representative areas on the surface of the ceramic.

Area	O	Si	K	Al	Na	C	Ba	Zr	S	Ca
(A)	47.84	20.96	4.61	6.05	4.73	14.80	-	-	-	1.01
(B)	47.94	12.21	2.84	3.25	3.06	24.83	3.26	-	2.61	-
(C)	24.21	9.59	0.99	1.23	2.08	61.90	-	-	-	-
(D)	44.72	8.41	2.77	2.57	2.01	34.47	-	5.05	-	-

## Data Availability

The raw data supporting the conclusions of this article will be made available by the authors on request.
